# The Association of Schools of Public Health in the European Region Statement on the Erosion of Public Health Systems

**DOI:** 10.3389/phrs.2021.1604112

**Published:** 2021-04-29

**Authors:** Brian L. H. Wong, Marine Delgrange, Naomi L. Nathan, Carolina D. Luévano, Jose M. Martin‐Moreno, Robert Otok, Ted H. Tulchinsky, John D. Middleton

**Affiliations:** ^1^ MRC Unit for Lifelong Health and Ageing at UCL, Department of Population Science and Experimental Medicine, Institute of Cardiovascular Science, London, United Kingdom; ^2^ COVID-19 Task Force, Association of Schools of Public Health in the European Region (ASPHER), Brussels, Belgium; ^3^ Department of Health Policy, London School of Economics and Political Science, London, United Kingdom; ^4^ Faculty of Public Health and Policy, London School of Hygiene and Tropical Medicine, University of London, London, United Kingdom; ^5^ École des Hautes Etudes en Santé Publique, Rennes, France; ^6^ Department of Preventive Medicine and INCLIVA, University of Valencia, Valencia, Spain; ^7^ Honours Committee, Association of Schools of Public Health in the European Region (ASPHER), Brussels, Belgium; ^8^ Secretariat, Association of Schools of Public Health in the European Region (ASPHER), Brussels, Belgium; ^9^ Braun School of Public Health and Community Medicine, Faculty of Medicine, Hebrew University of Jerusalem, Jerusalem, Israel; ^10^ Executive Board, Association of Schools of Public Health in the European Region (ASPHER), Brussels, Belgium

**Keywords:** public health, COVID-19, public health systems, global governance, global health security, global health, health systems, health systems strengthening

Public health (PH) systems are required to improve and protect the health of the people they serve. Over the past few decades, PH systems have eroded to the point where they cannot properly develop their four key functions–financing, provision, stewardship, and resource generation [1]–which allow essential PH operations to be carried out [2].

PH has suffered from major cuts in funding due to economic crises and government austerity policies. The financial crisis of 2008–9 and a rapidly aging global population have seen governments make efforts to reduce expenses and increase cost-effectiveness; the health sector in particular was an unfortunate target for such cuts. While in the 2000s, PH expenditure across the EU steadily increased, the reverse occurred when governments decreased health spending, resulting in increased out-of-pocket healthcare expenditures, weakened PH services, and exacerbated health inequalities globally [3, 4].

The decreasing ability of governments to generate sufficient resources is a key contributing factor to PH erosion. Health systems respond to limited budgets, and lower capacity amidst growing demand triggers a downward cycle of decreased access to and quality of care, resulting in higher burdens of disease. Moreover, the emergence of crises in underprepared and already strained systems consequently disrupts the services they usually deliver. This is not sustainable. Instead, long-term investment is essential to increase the resilience of health services so they can adequately face and recover from new challenges.

Deficiencies in the aforementioned functions also arise from a lack of vision, stewardship, and effective governance. Governments have either proven slow to listen to or have disregarded the advice of PH experts, sometimes even blaming the scientific community for failed responses. Not only does this contributes to the decreasing credibility of PH institutions, but it also leads to lack of public trust in politicians, impeding the success of measures which require buy-in from the entire population (e.g. vaccination compliance, social distancing, and mask wearing).

The extent to which PH systems have been eroded in many countries is particularly evident in the lack of swift and adequate response to COVID-19. The global response has highlighted gaps across a myriad of structures and inadequate preparedness and response mechanisms worldwide, a common thread in the failure to protect vulnerable populations [5, 6].

As the COVID-19 pandemic progressed, countries with well-functioning governance frameworks were able to rapidly increase and mobilize resources and demonstrated their capacity to test, isolate, trace, and quarantine.

Key factors underlying successes comprised:

- Improved preparedness and adaptability of PH systems following past experience (with SARS, MERS, Ebola) with prevention trainings and protocols in place [7].
- Strong collaboration between governments and the scientific community (e.g. Canada’s National Advisory Committee on SARS and PH) [8].
- Strong PH leadership embedded in policymaking, characterized by quick and decisive actions following WHO guidance [9].

By contrast, less effective national performance has resulted from:

- Failure to learn from evaluation reports, many of which illuminated the lack of preparedness and appropriate response frameworks [10].
- Lack of resources due to shrinking of regular public funding allocated to PH, which hindered quick and transparent deployment of additional funds and resources for crisis response;
- Failure to multi-agency network (e.g. with care homes, medical industry, media, etc … );
- Failure to listen to scientific advice and lack of dialogue between academia and policymakers [11].
- Governments falling into populist logics, encouraging skepticism in science and supremacy of personal freedoms over collective responsibility thinking [12].
- Lack of international leadership and collaboration in the spirit of the International Health Regulations.

In reviewing the pandemic response worldwide, a pattern of systemic and structural erosion of PH expertise and services emerges. Not only have susceptible communities been left unprotected, but the health and safety of “frontline” healthcare workers have also been compromised. Keeping health costs low or inappropriately allocating resources has and continues to cost human lives. In addition, delayed treatments, prolonged absence of preventive care, and lack of access to social and mental health services during lockdowns will contribute to increased morbidity and mortality [13].

Several elements point to the failure of many governments to respond swiftly and implement policies to combat infection. PH systems and services at all levels were in many cases under-prepared, undervalued, and under-resourced, and have occupied low priority for years. The lack of well-managed PH structures, clearly defined roles and responsibilities and funding cuts, all contributed to decreasing credibility and erosion of PH. Governments failed to recognize crucial interactions between PH and health services and the importance of the PH workforce (PHWF).

Amidst the pandemic, the United Kingdom government proposed to dissolve Public Health England (PHE) and replace it with a National Institute for Health Protection. This massive restructuring has left the PH community in further disarray. There have been some criticisms of PHE from professionals, but not for wholesale reorganization when efforts should have been focused on tackling the pandemic [14].

Moving forward, PH systems must be strengthened to ensure they are well-prepared to respond to pandemics and future PH challenges. ASPHER calls upon national governments to recognize PH as a vital component of national security and allocate more resources toward health systems strengthening (HSS). Governments should organize regular emergency preparedness training simulations according to WHO guidelines, followed by evaluation reports with realistic goals, to improve preparedness protocols. Additionally, governments must prioritize transparent and clear communication to the general public. Any measures taken should not only be detailed, harmonized, time-bound, and easily understandable, but also equitably enforced and regularly updated. Since PH lies at the crossroads of science and policy, continuous exchanges between decision makers and PH experts are essential to protect population health. Governments should not only include scientific experts in key national and regional decision-making committees, but also heed scientific advice and make recommendations based on the best evidence available.

PH organisations and the PHWF remain the backbone of all actions against pandemics and future health threats and they must be empowered to enact effective, agile, and responsive measures. Health systems need to ensure proper professionalization through continuous training and development of a highly skilled, diverse, and interdisciplinary PHWF, backed by strong PH policies and structures. Countries must ensure professionals are thoroughly trained in the full range of PH competencies and can function as part of a comprehensive PH system and service [15].

Given state/local authorities and many non-governmental organisations have PH responsibilities, they should be enabled to advocate for national governments to commit more resources and efforts toward HSS and emergency preparedness.

Associations, institutions, and schools should include health communication, stakeholder collaboration, and emergency preparedness in their global public health degrees as well as insert basic PH teaching into public administration and international affairs degrees. They should continue to develop PH professionals, while simultaneously training key healthcare personnel in infectious disease epidemiology competencies. Furthermore, they should advocate for appropriate investment in research into new health threats and effective responses.

International bodies and multilateral organisations should reinforce international collaboration toward HSS, paying particular attention to emergency preparedness, following international guidelines, and sharing best practices with national governments.

The COVID-19 pandemic has exposed the weaknesses of international and national health systems. As countries build back better [16], defining PH system needs, research agendas, and reinforcing international collaboration should be at the forefront. Moving forward, governments must prioritize PH and design long-term strategies aimed at strengthening health promotion and emergency preparedness measures to build sustainable health systems which make health for all a reality.

**Note:** An expanded statement can be found on ASPHER’s website here: https://www.aspher.org/download/675/aspher_ph_erosion_statement.pdf.
